# Systematic review of the use of behaviour change techniques (BCTs) in home-based cardiac rehabilitation programmes for patients with cardiovascular disease—protocol

**DOI:** 10.1186/s13643-015-0149-5

**Published:** 2015-11-17

**Authors:** Neil Heron, Frank Kee, Michael Donnelly, Mark A. Tully, Margaret E. Cupples

**Affiliations:** Centre for Public Health, School of Medicine, Dentistry and Biomedical Science, Queens University Belfast, Room 01012, Institute of Clinical Science B, Royal Victoria Hospital, Grosvenor Road, Belfast, BT12 6BA UK; UKCRC Centre of Excellence for Public Health (Northern Ireland), Institute of Clinical Science B, Royal Victoria Hospital, Grosvenor Road, Belfast, BT12 6BA UK

## Abstract

**Background:**

Cardiovascular diseases (CVDs), including myocardial infarction, heart failure, peripheral arterial disease and strokes, are highly prevalent conditions and are associated with high morbidity and mortality. Cardiac rehabilitation (CR) is an effective form of secondary prevention for CVD but there is a lack of information regarding which specific behaviour change techniques (BCTs) are included in programmes that are associated with improvements in cardiovascular risk factors. This systematic review will describe the BCTs which are utilised within home-based CR programmes that are effective at reducing a spectrum of CVD risk factors.

**Methods/design:**

The review will be reported in line with the preferred reporting items for systematic reviews and meta-analyses (PRISMA) guidance. Randomised and quasi-randomised controlled trials of home-based CR initiated following a vascular event (myocardial infarction, heart failure, peripheral arterial disease and stroke patients) will be included. Articles will be identified through a comprehensive search of MEDLINE, Embase, PsycINFO, Web of Science and Cochrane Database guided by a medical librarian. Two review authors will independently screen articles retrieved from the search for eligibility and extract relevant data, identifying which specific BCTs are included in programmes that are associated with improvements in particular modifiable vascular risk factors.

**Discussion:**

This review will be of value to clinicians and healthcare professionals working with cardiovascular patients by identifying specific BCTs which are used within effective home-based CR. It will also inform the future design and evaluation of complex health service interventions aimed at secondary prevention in CVD.

**Systematic review registration:**

PROSPERO registration CRD42015027036.

## Background

### Cardiovascular disease

Cardiovascular disease (CVD) is one of the leading causes of death, with survivors often being left with considerable morbidity and disability [1]. Cardiac rehabilitation is a very effective form of secondary prevention for CVD patients [2, 3]: it is a complex health service intervention with behaviour change techniques (BCTs) integral within its design aiming to deliver changes in different modifiable vascular risk factors. Indeed, Medical Research Council (MRC) guidelines [4, 5] advise the application of behaviour change theory within complex health service interventions to allow greater understanding of exactly how behaviour change is occurring. The use of behaviour change theory is reported to help both intervention design and evaluation [6].

### The National Institute of Health and Care Excellence guidelines

The National Institute of Health and Care Excellence (NICE) published guidance in 2014 on individual-level behaviour change interventions for promoting change in modifiable cardiovascular risk factors [7], building on previously published NICE guidance [8]. These guidelines indicated that the three BCTs most positively associated with promoting change in modifiable vascular risk factors were (1) goals and planning, (2) feedback and (3) monitoring and social support, which can be mapped to Michie’s BCT taxonomy [9].

### Cardiac rehabilitation

Cardiac rehabilitation for secondary prevention is offered to patients in the UK with cardiovascular disease [10]. The World Health Organization (WHO) has defined cardiac rehabilitation as the

“sum of activity and interventions required to ensure the best possible physical, mental and social conditions so that patients with chronic or post-acute cardiovascular disease may, by their own efforts, preserve or resume their proper place in society and lead an active life” [11].

NICE have recommended that the components of cardiac rehabilitation should include exercise, health education and stress management [10], helping to tackle vascular risk factors. Health education would include addressing the known modifiable vascular risk factors as well as advice regarding work, mental health and sexual activity [10]. These components are all addressed in the “Heart Manual”, the only validated home-based cardiac rehabilitation programme supported by the UK National Institute for Health and Clinical Excellence (NICE) for patients who have had a myocardial infarction (MI) [12].

Cardiac rehabilitation after a MI results in a statistically significant reduction in re-infarction (odds ratio 0.53), cardiac mortality (odds ratio 0.64), and all-cause mortality (odds ratio 0.74) [13] and these findings concur with those of a recent Cochrane Review which included other cardiovascular conditions [14]. Another Cochrane Review demonstrated that hospital- and home-based cardiac rehabilitation programmes, most of which used the Heart Manual, can result in similar health gains [2], with home-based programmes improving adherence to the programme [15]. Moreover, home-based cardiac programmes have shown longer-term sustainability of health benefits compared with hospital-based programmes [16].

Thus, there is strong evidence via meta-analysis and systematic reviews [2] to support the use of cardiac rehabilitation programmes, including home-based approaches, in a patient population with cardiovascular disease, particularly post-MI patients. Yet, despite this positive treatment option for vascular secondary prevention, there is not a clear understanding of how this complex health service intervention influences modifiable vascular risk factors. No previous review of the use of BCTs utilised in home-based cardiac rehabilitation programmes for cardiovascular patients, classified by using Michie’s BCT taxonomy [9], has been identified.

This review will thus be undertaken in order to identify the multiple BCTs used within home-based cardiac rehabilitation programmes and their association with change in modifiable vascular risk factors. The two primary research questions will be:

(i) What BCTs have been delivered to adults with cardiovascular disease within home-based cardiac rehabilitation programmes? and,
(ii) What specific BCTs are included in cardiac rehabilitation programmes which are associated with improvement in modifiable vascular risk factors?

### Why is it important to do this review?

Although rehabilitation and secondary prevention programmes following a cardiovascular event are theoretically well-evidenced [3], there is a paucity of evidence regarding which specific BCTs are associated with programmes that lead to improvement in particular cardiovascular risk factors, particularly within the setting of the patients’ home. This systematic review will help identify the particular BCTs which are associated with improvements in specific modifiable cardiovascular risk factors and therefore contribute to an evidence base upon which rehabilitation interventions can be further developed and refined for cardiovascular disease patients.

## Aim

This systematic review will identify the range of specific behaviour change techniques (BCTs) which are included in home-based cardiac rehabilitation programmes that are associated with improvements in specific modifiable cardiovascular risk factors.

### Key objectives

The key objectives of this study are to:

1. Identify home-based cardiac rehabilitation programmes which have been initiated following a cardiovascular event.
2. Determine the specific behaviour change techniques (BCTs) within home-based cardiac rehabilitation programmes, initiated following a cardiovascular event, which are associated with reductions in particular modifiable vascular risk factors.

## Methods/design

The behaviour change taxonomy v1 [9] will be used to identify the specific BCTs used within included studies. To ensure appropriate knowledge and understanding of this taxonomy, the lead authors have attended a training workshop by the taxonomy’s developers.

This systematic review will be reported in line with the preferred reporting items for systematic reviews and meta-analyses (PRISMA) guidance [17, 18]. Criteria for considering studies for this review will include the following:

### Types of studies

All human randomised and quasi-randomised controlled trials, published and unpublished, of home-based cardiac rehabilitation programmes for cardiovascular patients.

### Types of participants

The review will focus on adults 18 years of age or older that have received a diagnosis of cardiovascular disease and who are receiving home-based cardiac rehabilitation for secondary cardiovascular prevention. For clarity and to exclude more distinct populations, cardiovascular patients will include myocardial infarction, heart failure, peripheral arterial disease and cerebrovascular patients. No restrictions will be made based on gender.

### Types of interventions

Any rehabilitation programme or intervention delivered within the home environment aimed at tackling secondary prevention of vascular risk factors and events following an initial cardiovascular event will be eligible for inclusion, e.g. educational programmes, aerobic or exercise classes, self-management and lifestyle interventions. Trials will be included with comparative control groups and trials with multiple intervention arms, allowing comparison of different types of rehabilitation programmes. This study will not include population or community-wide interventions (e.g. mass media campaigns).

### Types of outcome measures

(i) Primary outcome
  To identify the particular behaviour change techniques included in cardiac rehabilitation programmes that are associated with the improvements in modifiable risk factors, including blood pressure, lipid profile (total cholesterol, high-density lipoprotein (HDL), low-density lipoprotein (LDL), triglycerides), glycaemic control in diabetes mellitus (HbA1c), body mass index (BMI) or validated cardiovascular risk score.
(ii) Secondary outcome
  To identify particular behaviour change techniques used in cardiac rehabilitation programmes that are associated with reductions in secondary cardiovascular events: stroke, myocardial infarction or vascular death.

### Search methods for identification of studies

Detailed search strategies will be developed for each electronic database searched with input from a medical librarian to allow identification of studies for inclusion in this review. The searches will be based on the strategy developed for Medical Literature Analysis and Retrieval System Online (MEDLINE) and revised appropriately.

### Electronic searches

The databases which will be searched will include the Ovid MEDLINE(R) 1946 to June 2015, Ovid Embase 1974 to June 2015, EBSCO Cumulative Index to Nursing and Allied Health Literature (CINAHL) plus 1937 to June 2015, Cochrane Database and Ovid PsycINFO 1806 to June 2015.

Any systematic reviews of rehabilitation interventions following a cardiovascular event (including myocardial infarction, heart failure, peripheral arterial disease and cerebrovascular disease) will be screened for additional references. Additional studies will be identified from reviewing the reference lists of the retrieved papers through a hand search.

### Study inclusion and exclusion criteria

The inclusion criteria are:

Adults aged 18 years old or above who have been diagnosed with a cardiovascular disease, including myocardial infarction, heart failure, peripheral arterial disease and cerebrovascular disease.

Assesses the impact of a home-based cardiac rehabilitation programme. Home-based rehabilitation programmes will be defined as per previous authors [2]:

“a structured programme, with clear objectives for the participants, including monitoring, follow-up, visits, letters, telephone calls from staff, or at least self monitoring diaries,”

delivered within the home environment.

The outcome measures for the study include a cardiovascular risk factor (e.g. blood pressure, physical activity levels), cardiovascular outcome (e.g. further stroke event) and/or death.

The study is a randomised controlled trial or quasi-randomised controlled trial.

The exclusion criteria are:

1. Includes cardiovascular patients outwith myocardial infarction, heart failure, peripheral arterial disease and cerebrovascular disease.

2. No rehabilitation service evaluation or evaluation of centre-based cardiac rehabilitation programme. As per previous authors [2], we defined centre-based rehabilitation programmes as:

“a supervised group based programme undertaken in a hospital or community setting such as a sports centre.”

These interventions are excluded from our study.

3. No modifiable CVD risk factors, cardiovascular events and/or death outcomes reported.

4. Protocol paper and therefore no results available.

### Data collection and analysis

#### Selection of studies

Results from the searches will be imported into a spread sheet and duplicates will be removed. The titles and abstracts of publications obtained from the search strategy will be independently screened by two authors (NH 100 %, MEC 100 %). Articles not meeting the inclusion criteria will be removed. Two review authors (NH, MEC) will use a standardised form as per previous authors [19] to select the trials eligible for inclusion. A third review author (FK) will resolve disagreements if required. A record will be kept of all articles excluded at this stage and the reason for their exclusion. The full papers will then be obtained for all studies remaining. No language restrictions will be made and appropriate arrangements will be made to translate non-English texts.

### Data extraction and management

Data from the studies will be extracted independently by the two review authors (NH, MEC) using an appropriate form. Data extracted will include information on methods, inclusion and exclusion criteria, type of interventions (including the behavioural change techniques involved [9]), study design and duration, follow-up, outcome measures, results, withdrawals and adverse events. The reviewers will meet to resolve any discrepancies, with third party adjudication if required.

Where there have been multiple publications of the same study, the team will try to extract and combine all of the available data and where there is doubt, the original publication will be given priority. If data seem to be missing from a study, we will try to obtain this through correspondence with the study authors. A table and flow diagram showing the characteristics of the included and excluded studies will be created. No blinding to study author, institution or journal will occur during the study screening process. The review team will resolve any disagreements regarding study eligibility by discussion between all review authors.

### Assessment of quality and risk of bias

The PEDrO scale [20] will be used to assess the quality of included papers. Two review authors (NH, MEC) will independently assess each included study for risk of bias (‘high’, ‘low’ or ‘uncertain’) using the risk of bias tool, following guidance from the Cochrane Handbook of Systematic Reviews of Interventions [21], a third review author (FK) will act as arbitrator as required.

### External validity

To ensure the results of the systematic review and meta-analysis are generalisable to the true study population of interest (those with a cardiovascular disease diagnosis), we will consider the external validity of all included studies. To do this, we will report on relevant aspects of the included studies in the review [22], including:

- Participant characteristics with reference to the background population (age, gender, source of recruitment);
- Sample size;
- Type and characteristics of the rehabilitation intervention;

This will allow us to comment on how representative the included studies are of the intended true population and therefore allow clinicians to better apply the evidence to their study population of interest.

### Missing data

We will try to contact the original investigators to request missing data.

### Assessing for heterogeneity

Diversity between the included studies will be assessed qualitatively in terms of the intervention (content, duration, frequency, provider, setting), participant demographics, outcome measures and duration of follow-up. If two or more studies are considered homogenous, data will be assessed for statistical heterogeneity using RevMan version 5.1 and the review team will use the chi-squared (χ2) test in conjunction with the I^2^ statistic. Where there is substantial heterogeneity, the review team will pool studies using a random effects model.

### Assessment of reporting bias

If there are sufficient studies, a funnel plot will be prepared to assess the effect of reporting and publication bias on our systematic review.

### Data synthesis

For complex healthcare interventions, effects can be modified by a wide array of factors. A narrative synthesis will be undertaken, including reviewing the behavioural change techniques used in the included studies [9].

## Discussion

Cardiac rehabilitation programmes, particularly home-based approaches, are well evidenced for secondary prevention in cardiovascular patients. To allow intervention optimisation, clinical trials need to apply theory throughout the design, implementation and evaluation stages of the intervention development. This will be the first systematic review to review the use of behaviour change techniques within home-based cardiac rehabilitation programmes for cardiovascular patients and their association with change in specific modifiable cardiovascular risk factors. The findings of this review may be applied in clinical and rehabilitation settings. The review will be of value to those involved in clinical research, including those involved in the design, development and implementation of complex health service interventions, particularly for cardiovascular patients, by helping to identify which BCTs are associated with reductions in individual modifiable vascular risk factors and secondary vascular event prevention.

## Abbreviations

BCTs: behaviour change techniques
BMI: body mass index
CR: cardiac rehabilitation
CVD: cardiovascular disease
HbA1c: glycated haemoglobulin
HDL-cholesterol: high-density lipoprotein cholesterol
LDL-cholesterol: low-density lipoprotein cholesterol
MRC: Medical Research Council (UK)
MI: myocardial infarction
NICE: National Institute of Health and Care Excellence
NIHR: National Institute of Health Research
PEDro scale: Physiotherapy Evidence Database scale
PRISMA guidance: preferred reporting items for systematic reviews and meta-analyses guidance
WHO: World Health Organization

## References

[1] World Health Organisation (WHO). Global health observatory data repository. World Health Organisation (WHO) 2014;apps.who.int/gho/data/ node.main (accessed 7 December 2014).

[2] Dalal HM, Zawada A, Jolly K, Moxham T, Taylor RS (2010). Home based versus centre based cardiac rehabilitation: Cochrane systematic review and meta-analysis. Br Med J..

[3] Anderson L, Taylor R (2014). Cardiac rehabilitation for people with heart disease: an overview of Cochrane systematic reviews (review). Cochrane Database Syst Rev.

[4] Craig P, Dieppe P, Macintyre S, Michie S, Nazareth I, Petticrew M (2008). Developing and evaluating complex interventions: the new Medical Research Council guidance. Br Med J.

[5] Craig P, Dieppe P, MacIntyre S, Michie S, Nazareth I, Petticrew M (2013). Developing and evaluating complex interventions: The new Medical Research Council guidance. Int J Nurs Stud.

[6] Davis R, Campbell R, Hildon Z, Hobbs L, Michie S (2014). Theories of behaviour and behaviour change across the social and behavioural sciences: a scoping review. Health Psychol Rev..

[7] National Institute for Health and Care Excellence (NICE). Behaviour change: individual approaches. NICE Public Health Guidance. 2014;49.

[8] National Institute of Health and Care Excellence (NICE). Behaviour change: the principles for effective interventions. NICE Public Health Guidance. 2007;6.

[9] Michie S, Richardson M, Johnston M, Abraham C, Francis J, Hardeman W (2013). The behavior change technique taxonomy (v1) of 93 hierarchically clustered techniques: building an international consensus for the reporting of behavior change interventions. Ann Behav Med.

[10] National Institute of Health and Clinical Excellence. CG48 MI; secondary prevention. 2010.

[11] Report of a WHO Expert Committee. Rehabilitation after cardiovascular-diseases, with special emphasis on developing-countries. World Health Organ Tech Rep Ser. 1993;831:1-122. 8351937

[12] National Institute of Health and Clinical Excellence (2007). Secondary prevention in primary and secondary care for patients following a myocardial infarction.

[13] Lawler PR, Filion KB, Eisenberg MJ (2011). Efficacy of exercise-based cardiac rehabilitation post–myocardial infarction: a systematic review and meta-analysis of randomized controlled trials. Am Heart J.

[14] Heran BS, Chen JMH, Ebrahim S, Moxham T, Oldridge N, Rees K (2011). Exercise-based cardiac rehabilitation for coronary heart disease. Cochrane Database Syst Rev.

[15] Clark AM, Haykowsky M, Kryworuchko J, MacClure T, Scott J, DesMeules M (2010). A meta-analysis of randomized control trials of home-based secondary prevention programs for coronary artery disease. Eur J Cardiovasc Prevent Rehabil.

[16] Smith K, Arthur H, McKelvie R, Kodis J (2004). Differences in sustainability of exercise and health-related quality of life outcomes following home or hospital-based cardiac rehabilitation. Eur J Cardiovasc Prevent Rehabil.

[17] Ferrier S, Blanchard CM, Vallis M, Giacomantonio N (2011). Behavioural interventions to increase the physical activity of cardiac patients: a review. Eur J Cardiovasc Prevent Rehabil.

[18] Keogh A, Tully M, Matthews J, Hurley D. A review of behaviour change theories and techniques used in group based self-management programmes for chronic low back pain and arthritis. Manual Ther. 2015.(Pub ahead of print). 10.1016/j.math.2015.03.014 25865062

[19] Marley J, Tully M, Porter-Armstrong A, Bunting B, O’Hanlon J, McDonough S (2014). A systematic review of interventions aimed at increasing physical activity in adults with chronic musculoskeletal pain—protocol. Syst Rev.

[20] Maher CG, Sherrington C, Herbert RD, Moseley AM, Elkins M (2003). Reliability of the PEDro scale for rating quality of randomized controlled trials. Phys Ther.

[21] Higgins J, Green S. (Cochrane Handbook for Systematic Reviews of Interventions Version 5.1.0 [updated March 2011]. Cochrane Collaboration. 2011

[22] Nasser M, van Weel C, van Binsbergen JJ, van de Laar FA (2012). Generalizability of systematic reviews of the effectiveness of health care interventions to primary health care: concepts, methods and future research. Fam Pract.

